# Clinical remission trajectories and histopathological associations in patients with IgA nephropathy treated with targeted-release budesonide: a real-world cohort study

**DOI:** 10.3389/fimmu.2026.1874529

**Published:** 2026-09-03

**Authors:** Dafeng He, Jun Xu, Bo Gao, Mingzhu Jiang, Wenjin Liu, Chuanyan Zhao, Xinrui Li, Chunlei Lu, Mengyue Zhu, Hongbin Mou, Guangyu Bi

**Affiliations:** Nephrology Department, Northern Jiangsu People’s Hospital Affiliated to Yangzhou University, Yangzhou, Jiangsu, China

**Keywords:** IgA nephropathy, immune-inflammatory phenotype, mucosa-kidney axis, prognostic evaluation, prognostic nomogram, remission kinetics, targeted-release budesonide

## Abstract

**Background:**

Targeted-release (TR) budesonide provides upstream mucosal immunomodulation in IgA nephropathy (IgAN) by targeting the gut-associated lymphoid tissue. However, the extent to which distinct downstream immune-inflammatory phenotypes and established structural chronicity shape real-world remission trajectories remains insufficiently characterized.

**Methods:**

We retrospectively analyzed 57 adults with biopsy-proven primary IgAN treated with TR-budesonide. Prespecified milestones were Very Early Response (VER) at 3 months, Early Response (ER) at 6 months, and composite clinical remission (complete or partial remission, CR/PR) over 12 months. We constructed a time-dependent prognostic nomogram using multivariable Cox modeling integrating the full Oxford MEST-C spectrum and concurrent therapies, strictly applying optimism-corrected bootstrapping (1,000 iterations) to ensure robust internal validation.

**Results:**

Relative proteinuria reductions were broadly comparable across MEST-C strata, even among patients with substantial baseline chronicity (S1, 91.2%; T1/T2, 63.2%). Concomitant sodium–glucose cotransporter 2 inhibitor (SGLT2i) therapy independently predicted VER (OR 9.40, 95% CI 1.36–64.92; P = 0.023), consistent with an early complementary kinetic profile. Over 12 months, endocapillary hypercellularity (E1) was the only independent histopathological factor associated with composite remission (OR 2.20; P = 0.022) and was associated with a shorter time to remission (log-rank P = 0.031). The resulting optimized 3-variable nomogram demonstrated good discrimination (time-dependent AUC 0.79 at 6 months and 0.83 at 12 months) with favorable internal calibration.

**Conclusions:**

In routine practice, the antiproteinuric effect of TR-budesonide appears largely preserved even in patients with advanced tubulointerstitial fibrosis. Early response is strongly associated with concomitant SGLT2i therapy, while the E1 lesions was associated with a faster trajectory toward composite clinical remission within this treated cohort, identifying a potentially reversible inflammatory phenotype that warrants further validation. These findings highlight the translational value of integrating histopathological phenotyping to characterize real-world remission trajectories.

## Highlights

What was known:

- Phase 3 trials established that targeted-release (TR) budesonide improves renal outcomes in IgA nephropathy by suppressing intestinal mucosal immunity and modulating the mucosa-kidney axis.
- However, the extent to which established downstream structural chronicity limits the benefits of this upstream immunomodulation remains poorly characterized in real-world practice.
- Furthermore, the translational relevance of specific immune-inflammatory phenotypes (such as active endocapillary inflammation, E1) and the kinetic impact of combining immunomodulation with downstream hemodynamic therapies (e.g., SGLT2 inhibitors) are largely unknown.

This study adds:

- Proportional proteinuria reduction was observed across the diverse MEST-C spectrum, including in patients with tubulointerstitial fibrosis, though these trajectories reflect combined real-world regimens.
- Active endocapillary hypercellularity (E1) emerges as the sole independent pathological factor associated with long-term composite clinical remission, identifying a specific, potentially reversible immune-inflammatory phenotype.
- Concomitant SGLT2 inhibition provides a rapid hemodynamic benefit, independently driving very early clinical response before the full onset of long-term clinical remission.

Potential impact:

- These findings suggest that advanced downstream structural chronicity does not inherently preclude favorable antiproteinuric responses to TR-budesonide.
- Identifying the E1 lesion as a phenotype associated with rapid kinetics provides a novel translational framework for evaluating accelerated healing kinetics.
- Our internally validated, time-dependent nomogram integrates these complex clinico-pathological variables into exploratory remission probability estimates in IgA nephropathy.

## 1 Introduction

Immunoglobulin A nephropathy (IgAN) is driven by dysregulated communication between gut-associated lymphoid tissue (GALT) and the renal microvasculature, a process commonly referred to as the mucosa-kidney axis (1, 2). In the established multi-hit model, disease initiation is linked to persistent mucosal overproduction of galactose-deficient IgA1 (Gd-IgA1), which promotes downstream immune complex formation and glomerular injury (1, 3). Clinical management has historically been largely reactive, focusing on mitigation of secondary hemodynamic and fibrotic injury rather than interruption of upstream immune drivers (4). Although optimized renin–angiotensin system (RAS) blockade (5) and sodium–glucose cotransporter 2 inhibitors (SGLT2i) reduce intraglomerular pressure and slow progression by limiting maladaptive remodeling, these approaches do not directly target the initiating mucosal immunopathology (6, 7). Targeted-release (TR) budesonide was developed to address this upstream pathway. Formulated to release in the terminal ileum, TR-budesonide acts on Peyer’s patches to suppress mucosal immune activation and reduce the production of pathogenic Gd-IgA1 (8, 9). Extensive hepatic first-pass metabolism limits systemic exposure and reduces the risk of adverse effects typically associated with conventional systemic corticosteroids (8). The phase 3 NefIgArd trial demonstrated that TR-budesonide lowers proteinuria and preserves estimated glomerular filtration rate (eGFR) (10, 11), supporting its incorporation into updated guideline-based strategies as a disease-modifying therapy for IgAN (6). Despite these advances, important uncertainties remain when applying trial evidence to routine practice, particularly given the clinico-pathological heterogeneity captured by the Oxford MEST-C classification (12, 13). First, patients with advanced structural chronicity, including tubulointerstitial fibrosis (T lesions) and segmental glomerulosclerosis (S lesions), are often considered suboptimal candidates for systemic immunosuppression because irreversible damage may limit benefit and increase risk (14, 15). Whether TR-budesonide shows favorable clinical remission trajectories and renal function preservation in such highly chronic phenotypes in real-world settings remains to be carefully explored. Second, the clinical relevance of active inflammatory lesions for treatment timing remains uncertain. Endocapillary hypercellularity (E1) reflects active mesangial–endothelial injury and ongoing inflammation (16, 17), but its utility as a marker of a treatment-responsive window for upstream mucosal intervention requires validation outside trial populations. Third, the early response kinetics of combining upstream mucosal immunomodulation with downstream hemodynamic therapy have not been quantified. In practice, TR-budesonide is frequently co-administered with SGLT2i, raising the possibility that dual targeting of immune and hemodynamic pathways may accelerate early response. Finally, although tools such as the International IgAN Risk Prediction Tool estimate baseline prognosis under standard care (18), clinicians lack therapy-specific instruments that translate baseline pathology and concomitant treatments into individualized remission expectations for targeted interventions.

To address these gaps, we sought to explore the real-world therapeutic kinetics of TR-budesonide across the full spectrum of Oxford MEST-C phenotypes. We aimed to examine whether favorable clinical remission and renal trajectories are observed in chronic lesions, identify histopathological markers of accelerated remission with particular attention to E1, and assess how concomitant SGLT2i therapy relates to early response. We further developed an internally validated and exploratory, time-dependent prognostic nomogram to support individualized counseling and shared decision-making frameworks for patients receiving TR-budesonide, pending external validation.

## 2 Materials and methods

### 2.1 Study design and participants

**Figure 1:**
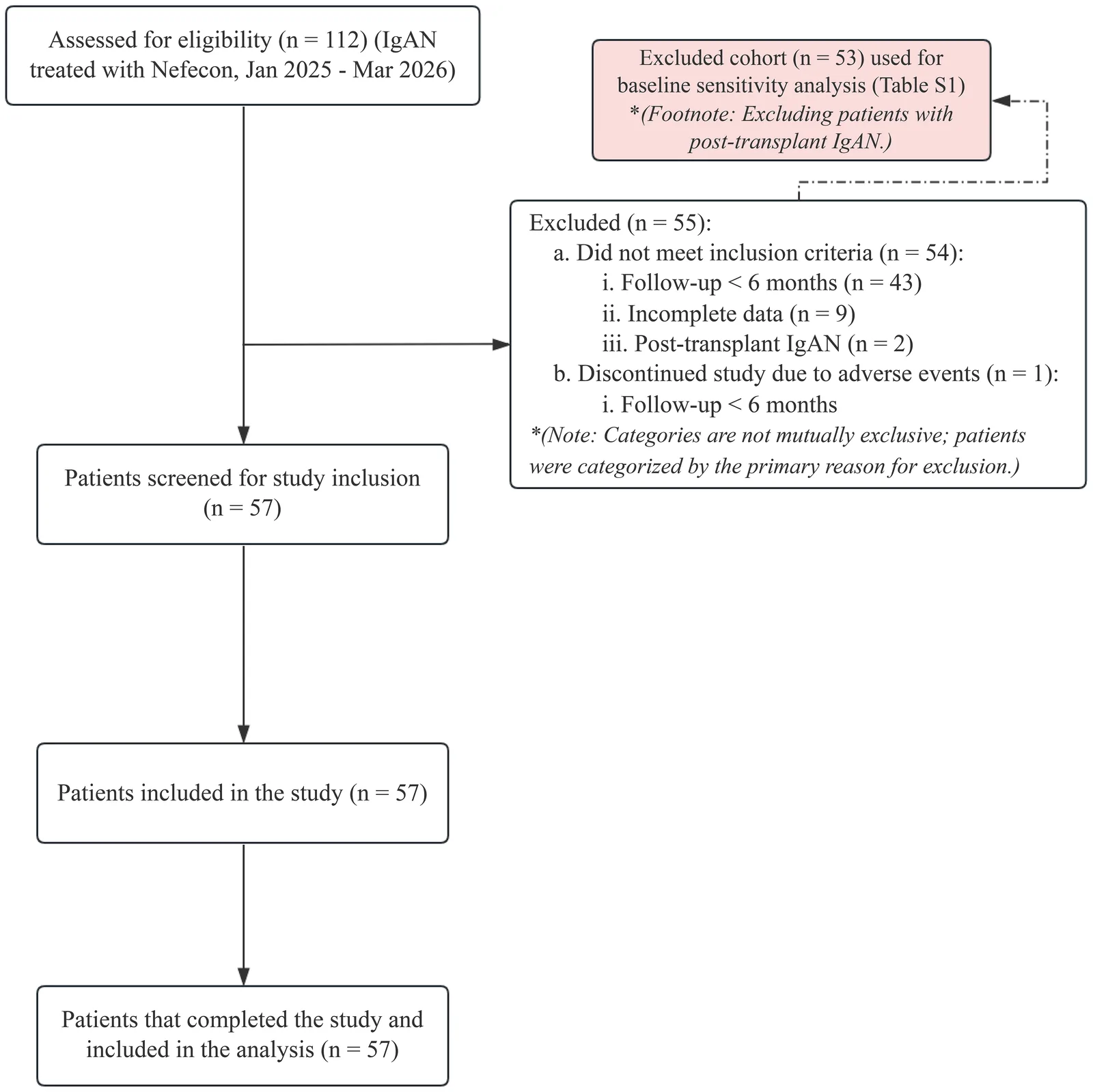
Patient selection and cohort stratification. Screening and enrollment workflow in accordance with STROBE recommendations. A total of 112 patients with IgAN were assessed for eligibility. Among them, 55 patients were excluded: 54 did not meet the predefined inclusion criteria, and 1 discontinued due to adverse events. Within the excluded group, 53 patients (excluding those with post-transplant IgAN) were included in the baseline sensitivity analysis (**Supplementary Table 1**). A total of 57 patients were ultimately included in the final analysis. *IgAN, immunoglobulin A nephropathy; MMF, mycophenolate mofetil; SGLT2i, sodium-glucose cotransporter 2 inhibitor*.

This retrospective observational cohort study was conducted in accordance with the STROBE statement (19) and the Declaration of Helsinki. The study protocol was reviewed and approved by the local institutional review board (Approval No. 2026KY167). We screened adults (aged 18 years or older) with biopsy-proven primary IgA nephropathy (IgAN) who initiated targeted-release (TR) budesonide (Nefecon) at our center. Eligibility required a baseline estimated glomerular filtration rate (eGFR) of at least 15 mL/min/1.73 m² and sufficient longitudinal follow-up to evaluate prespecified therapeutic milestones. We excluded patients with secondary IgA nephropathy, those without complete baseline Oxford MEST-C scoring, and those with follow-up shorter than 6 months. The screening and enrollment process is summarized in **Figure 1**. To evaluate potential selection bias, baseline demographics, clinical parameters, and Oxford MEST-C scores were compared between the analyzed cohort (N = 57) and the excluded cohort (N = 53) (**Supplementary Table S1**).

### 2.2 Clinicopathological evaluation

Baseline demographics, laboratory indices, and concomitant medications were extracted from electronic medical records at the time of TR-budesonide initiation, including maximally tolerated renin–angiotensin system (RAS) inhibitors, sodium–glucose cotransporter 2 inhibitors (SGLT2i), and mycophenolate mofetil (MMF), as applicable. Proteinuria was assessed using either 24-hour urine protein excretion (g/24 h) or the spot urine protein-to-creatinine ratio (UPCR, g/gCr). When both measures were available at a given time point, 24-hour collection was prioritized. For analytical harmonization and in line with established clinical trial consensus for IgA nephropathy (20), UPCR was utilized as a validated surrogate for 24-hour urine protein excretion, applying the standard conversion rule where 1 g/gCr mirrors 1 g/24 h (methodological assay distribution detailed in **Supplementary Table 2**). Renal biopsies were reviewed by experienced renal pathologists who were blinded to clinical outcomes. Histopathology was graded using the Oxford MEST-C classification: mesangial hypercellularity (M0/M1), endocapillary hypercellularity (E0/E1), segmental glomerulosclerosis (S0/S1), tubulointerstitial atrophy and interstitial fibrosis (T0/T1/T2), and cellular or fibrocellular crescents (C0/C1/C2) (12, 13).

### 2.3 Clinical endpoints and kinetic definitions

We defined prespecified temporal milestones to characterize treatment kinetics. The primary early endpoints were Very Early Response (VER) at 3 months and Early Response (ER) at 6 months. Both were defined as a 30% or greater proportional reduction in proteinuria from baseline, measured by either 24-hour urine protein or UPCR, together with functional stabilization defined as an eGFR decline of less than 15% from baseline (20). The long-term endpoint was Composite Clinical Remission over a 12-month observation period, defined as complete remission (CR) or partial remission (PR) with concomitant eGFR stability. CR was defined as proteinuria below 0.3 g/day, whereas PR was defined as a 50% or greater reduction in proteinuria with an absolute value below 3.5 g/day. Both CR and PR required an eGFR decline of less than 15% from baseline. Because the observation window reflects routine follow-up for a newly implemented targeted therapy, we prioritized categorical eGFR stability rather than short-term eGFR slope estimation. This approach reduces bias introduced by early, reversible hemodynamic changes (the eGFR dip) commonly observed after initiation of SGLT2i or RAS inhibition (21, 22).

### 2.4 Global standardization of immune-inflammatory indices

Baseline systemic immune-inflammatory indices were computed from peripheral blood counts, including the neutrophil-to-lymphocyte ratio (NLR), platelet-to-lymphocyte ratio (PLR), systemic immune-inflammation index (SII), systemic inflammation index (SIAI), and neutrophil-to-high-density lipoprotein ratio (NHR) (18, 23). To facilitate comparability of effect estimates across models, each index was standardized using a Z-score transformation before regression analyses. Standardization parameters (mean and standard deviation) were derived from the entire baseline cohort to ensure that each 1-standard deviation increase reflected the relative inflammatory burden within the study population (**Supplementary Table 3**).

### 2.5 Statistical analysis and predictive modeling

Continuous variables are reported as mean ± standard deviation (SD) or median with interquartile range (IQR), as appropriate. Between-group comparisons were performed using Student’s t test, the Mann-Whitney U test, or the chi-square test or Fisher’s exact test, depending on data distribution and cell counts. Longitudinal trajectories of proteinuria and eGFR were evaluated using mixed-effects models. Time-to-event analyses for composite remission were performed using Kaplan–Meier methods, and group differences were assessed with the log-rank test. Multivariable logistic regression and Cox proportional hazards models were used to identify independent predictors of early categorical milestones (VER and ER) and composite remission, respectively. The proportional hazards assumption for Cox models was evaluated using Schoenfeld residuals. To reduce data-driven selection bias, we applied an *a priori* covariate inclusion strategy (24). Variable selection was performed via LASSO-penalized Cox regression, yielding a parsimonious 3-variable model comprising baseline proteinuria, E1 status, and concomitant SGLT2i therapy. Given the exploratory nature of secondary subgroup comparisons and interaction assessments, all corresponding P values were evaluated using nominal significance thresholds. Multicollinearity was assessed using a Spearman correlation matrix, which did not indicate severe interdependence among candidate predictors (**Supplementary Figure 4**). A prognostic nomogram was constructed from the final multivariable Cox model. Model performance was evaluated by discrimination using time-dependent receiver operating characteristic analysis with area under the curve (AUC) estimates at 6 and 12 months (25), calibration using optimism-corrected bootstrap resampling (1,000 iterations), and clinical utility using decision curve analysis (26). Statistical analysis and predictive modeling were performed using a dual-software approach to ensure robust computational execution. Analytical procedures were executed using Python (version 3.8 or higher) and R software (version 4.2.0), and a two-sided P value below 0.05 was considered statistically significant.

## 3 Results

### 3.1 Baseline clinicopathological characteristics

A total of 57 adults treated with targeted-release (TR) budesonide were included in the final analytic cohort. Baseline characteristics are summarized in **Table 1**. The mean age was 40.9 ± 8.3 years, the median baseline proteinuria was 1.21 g/day (IQR 0.60 to 2.38), and the mean baseline eGFR was 61.3 ± 25.2 mL/min/1.73 m². Histopathology demonstrated a substantial burden of chronic structural injury, with segmental glomerulosclerosis (S1) present in 91.2% of patients and moderate-to-severe tubulointerstitial atrophy and fibrosis (T1/T2) in 63.2%. Active inflammatory lesions were also common, including mesangial hypercellularity (M1) in 66.7% and endocapillary hypercellularity (E1) in 49.1%. There were no statistically significant differences in baseline demographics, renal function (including eGFR and proteinuria), or Oxford MEST-C pathological classifications between the analyzed and excluded cohorts (all P > 0.05; detailed in **Supplementary Table 1**). This indicates that patient attrition was random relative to baseline disease severity.

**Table 1:** Baseline demographic and clinical characteristics of the study cohort (N = 57).

Characteristics	Overall (N = 57)
Demographics and clinical comorbidities	
Age (years), mean ± SD	40.9 ± 8.3
Male sex, n (%)	32 (56.1%)
Body mass index (kg/m²), median (Q1–Q3)	23.5 (20.3–25.1)
Hypertension, n (%)	15 (26.3%)
Diabetes mellitus, n (%)	2 (3.5%)
Baseline laboratory parameters	
eGFR (mL/min/1.73m²), mean ± SD	61.3 ± 25.2
eGFR stratification, n (%)	
≥ 60 mL/min/1.73m²	29 (50.9%)
< 60 mL/min/1.73m²	28 (49.1%)
Serum creatinine (μmol/L), median (Q1–Q3)	119.0 (93.0–142.0)
Proteinuria (g/d or g/g.Cr), median (Q1–Q3)	1.21 (0.60–2.38)
Proteinuria stratification, n (%)	
< 0.5 g/24h, or g/g.Cr	9 (15.8%)
0.5–1.99 g/24h, or g/g.Cr	30 (52.6%)
2.0–3.49 g/24h, or g/g.Cr	10 (17.5%)
≥ 3.5 g/24h, or g/g.Cr	8 (14.0%)
Microhematuria at baseline, n (%)	
Oxford classification (MEST-C score), n (%)	
M1 (Mesangial hypercellularity)	38 (66.7%)
E1 (Endocapillary hypercellularity)	28 (49.1%)
S1 (Segmental glomerulosclerosis)	52 (91.2%)
T1/T2 (Tubular atrophy/interstitial fibrosis)	24 (42.1%)/12 (21.1%)
C1 (Cellular/fibrocellular crescents)	22 (38.6%)
Previous immunosuppressive and targeted therapies, n (%)	
Glucocorticoids	13 (22.8%)
Mycophenolate mofetil (MMF)	6 (10.5%)
Hydroxychloroquine (HCQ)	19 (33.3%)
SGLT2 inhibitors	19 (33.3%)
Concomitant medications at baseline, n (%)	
RAS inhibitors (ACEI/ARB)	30 (52.6%)
SGLT2 inhibitors	34 (59.6%)
Glucocorticoids	13 (22.8%)
Mycophenolate mofetil (MMF)	10 (17.5%)
Hydroxychloroquine (HCQ)	13 (22.8%)

Continuous variables are presented as mean ± standard deviation (SD) when normally distributed and as median (interquartile range [IQR], Q1 to Q3) when non-normally distributed, as assessed using the Shapiro–Wilk test. Categorical variables are reported as counts (percentages).

eGFR, estimated glomerular filtration rate; RAS, renin-angiotensin system; ACEI, angiotensin-converting enzyme inhibitor; ARB, angiotensin II receptor blocker; SGLT2, sodium-glucose cotransporter-2.

### 3.2 Longitudinal proteinuria reduction and eGFR stabilization

**Figure 2:**
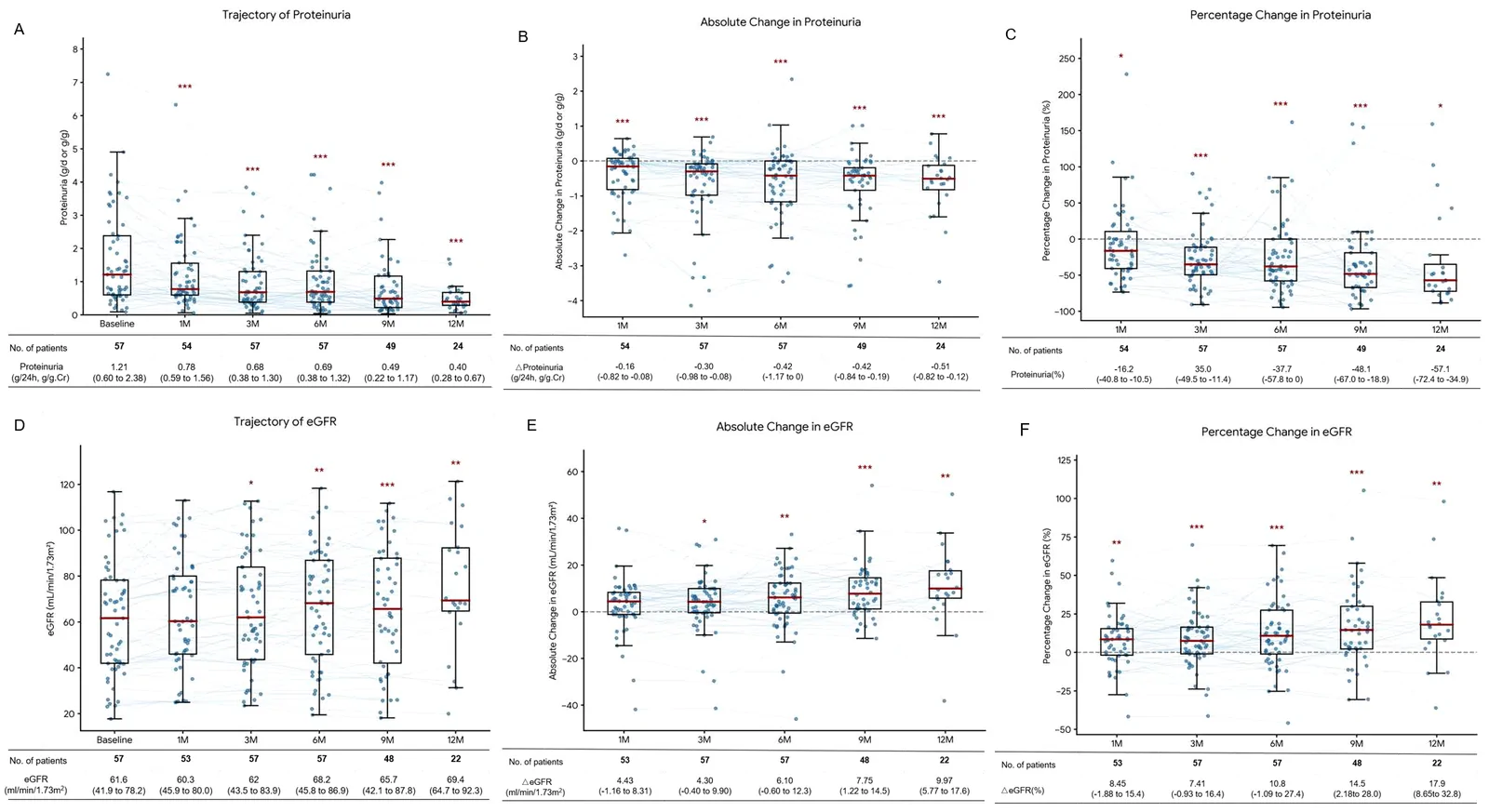
Longitudinal kinetics of significant proteinuria reduction and preservation of renal function. **(A)** Absolute changes in 24-hour urine protein excretion (g/day). **(B)** Percentage change in proteinuria relative to baseline. **(C)** Shifts in categorical proteinuria strata over time. **(D)** Absolute change in estimated glomerular filtration rate (eGFR; mL/min/1.73 m²) from baseline. **(E)** Percentage change in eGFR relative to baseline. **(F)** Distribution of categorical eGFR trajectories, including the proportion of patients with stable renal function (defined as eGFR within ±15% of baseline). Data are presented as mean ± SD or median (IQR), as appropriate. Longitudinal changes and between-group comparisons were evaluated using linear mixed-effects models and non-parametric tests, as appropriate. eGFR, estimated glomerular filtration rate; IQR, interquartile range; SD, standard deviation. *, **, and *** represent P<0.05, P<0.01, and P<0.001, respectively.

TR-budesonide treatment was associated with sustained improvements in proteinuria and preservation of kidney function. Longitudinal analyses showed significant reductions in absolute 24-hour proteinuria (**Figure 2A**), consistent proportional reductions from baseline (**Figure 2B**), and favorable shifts in categorical proteinuria strata over time (**Figure 2C**). As detailed in **Supplementary Table 2**, median proteinuria decreased from 1.21 g/day at baseline to 0.40 g/day at 12 months, corresponding to a 57.1% relative reduction. Renal function remained generally stable over follow-up. Absolute eGFR changes (**Figure 2D**), relative eGFR trajectories (**Figure 2E**), and categorical eGFR distributions (**Figure 2F**) showed no evidence of progressive functional decline at the cohort level. Mean eGFR increased from 61.3 ± 25.2 mL/min/1.73 m² at baseline to 74.9 ± 27.5 mL/min/1.73 m² at 12 months (**Supplementary Table 5**). To exclude the possibility that these favorable longitudinal trajectories were driven by informative attrition bias, a complete-case sensitivity analysis was conducted in patients with a full 12-month prospective record (n=22). The clinical trends in this stable sub-cohort mirrored the primary efficacy analysis, confirming the robustness of the functional stabilization (**Supplementary Table 6**). Regarding safety and tolerability, TR-budesonide demonstrated a favorable profile. Across the entire screened population (n=112), mild cushingoid features (moon face) occurred in 3.57% of patients, and no severe opportunistic infections were recorded. As detailed in the screening flowchart (**Figure 1**), only 1 patient (0.89%) discontinued treatment due to severe hypertension. Full safety registries are provided in **Supplementary Table 7**, noting that nearly all encountered adverse events were mild (Grade 1–2) and resolved without systemic complications. Of note, while safety outcomes were audited across the entire screened registry (n = 112), efficacy evaluations were strictly restricted to the analyzed cohort (n = 57).

### 3.3 Response patterns across baseline MEST-C pathology

**Figure 3:**
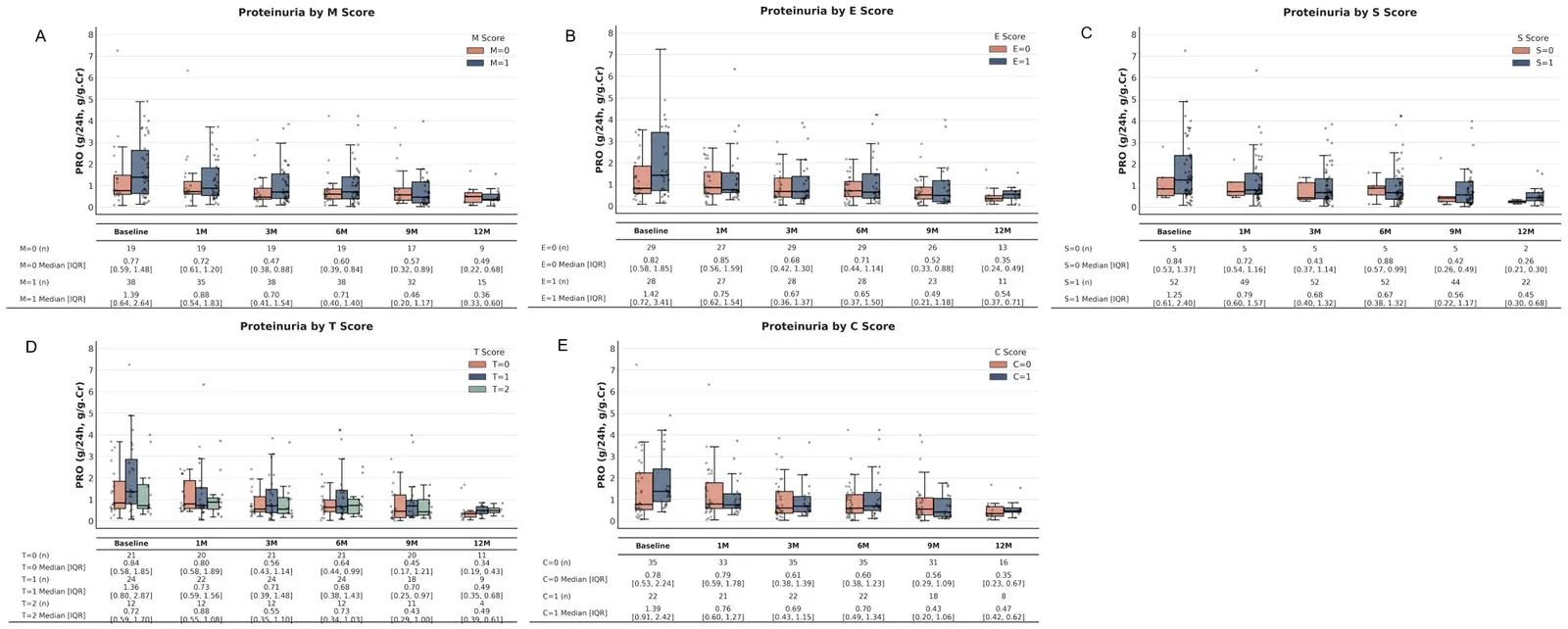
Proteinuria reduction stratified by baseline MEST-C pathology. Percentage reduction in proteinuria from baseline stratified by individual MEST-C components: **(A)** mesangial hypercellularity (M0 vs M1), **(B)** endocapillary hypercellularity (E0 vs E1), **(C)** segmental glomerulosclerosis (S0 vs S1), **(D)** tubulointerstitial atrophy and interstitial fibrosis (T0 vs T1/T2), and **(E)** cellular or fibrocellular crescents (C0 vs C1/C2). Between-group comparisons across time points were performed using the Mann–Whitney U test (two groups) or the Kruskal–Wallis test (three or more groups). *eGFR, estimated glomerular filtration rate; IQR, interquartile range; MEST-C, Oxford pathology score*.

**Figure 4:**
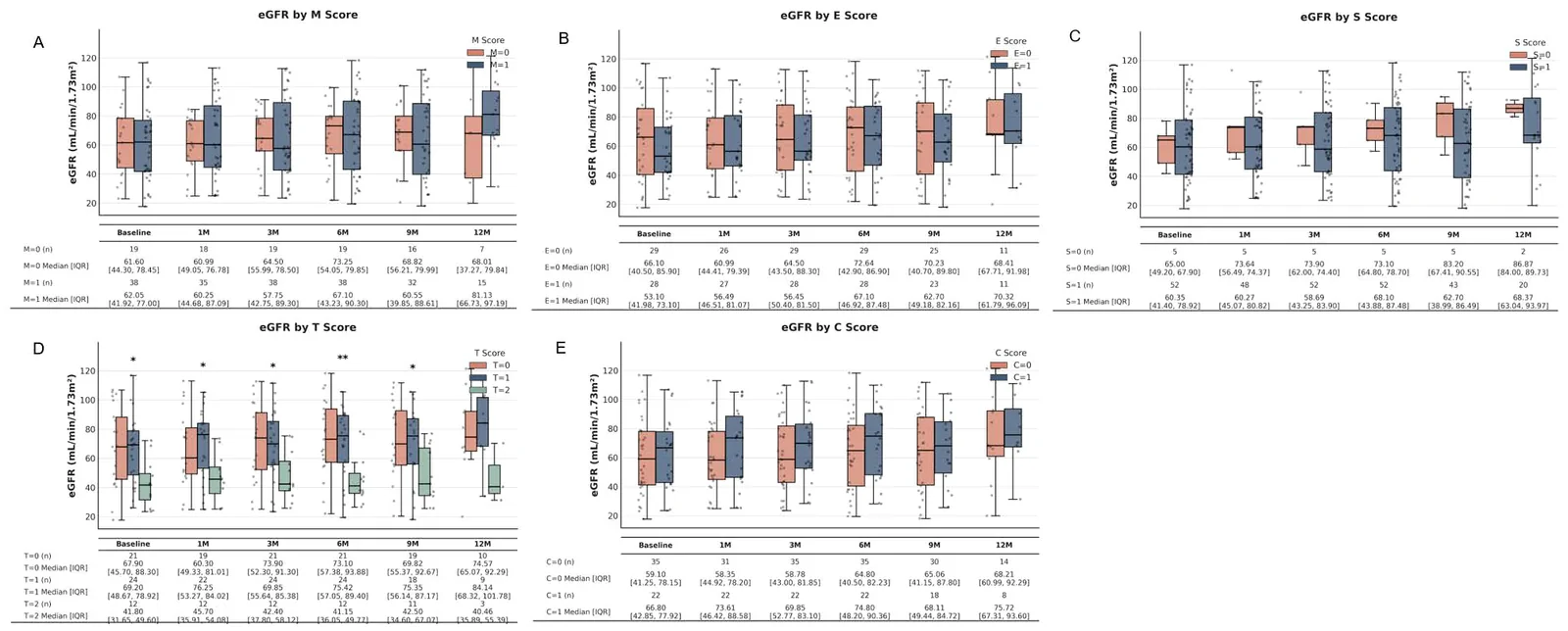
eGFR trajectories stratified by baseline MEST-C pathology. Absolute eGFR values over time stratified by individual MEST-C components: **(A)** mesangial hypercellularity (M0 vs M1), **(B)** endocapillary hypercellularity (E0 vs E1), **(C)** segmental glomerulosclerosis (S0 vs S1), **(D)** tubulointerstitial atrophy and interstitial fibrosis (T0 vs T1/T2), and **(E)** cellular or fibrocellular crescents (C0 vs C1/C2). Data are shown as median (IQR). Between-group comparisons were conducted using the Mann-Whitney U test or the Kruskal–Wallis test, as appropriate. eGFR, estimated glomerular filtration rate; IQR, interquartile range; MEST-C, Oxford pathology score.

We evaluated treatment trajectories after stratification by individual Oxford MEST-C components. Relative proteinuria reductions appeared broadly consistent across subgroups, indicating that the proportional antiproteinuric response was not materially modified by baseline mesangial hypercellularity (**Figure 3A**), endocapillary hypercellularity (**Figure 3B**), segmental glomerulosclerosis (**Figure 3C**), tubulointerstitial fibrosis (**Figure 3D**), or crescentic lesions (**Figure 3E**); however, given the high prevalence of S1 lesions, findings within the sparse S0 reference group should be interpreted with appropriate caution. Consistent with these findings, eGFR remained largely stable in patients with active inflammatory lesions (M1 and E1; **Figures 4A, B**) and in those with chronic S1 lesions (**Figure 4C**). As expected, patients with advanced tubulointerstitial fibrosis (T1/T2) had lower absolute eGFR values throughout follow-up, reflecting established nephron loss rather than treatment-related decline (**Figure 4D**). eGFR trajectories did not differ significantly over time across crescent strata (C0 vs C1/C2; **Figure 4E**).

### 3.4 Predictors of early clinical milestones and profiles of concomitant therapies

**Figure 5:**
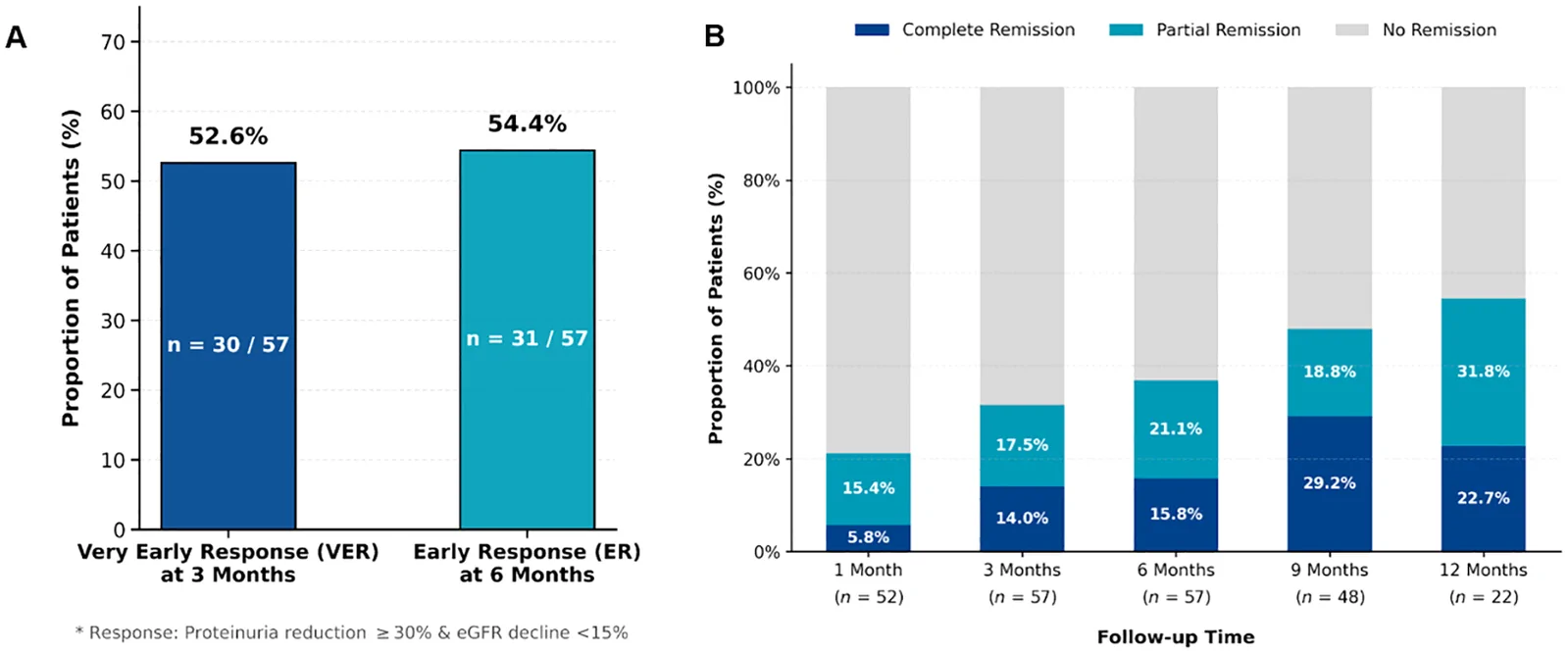
Early clinical milestones and longitudinal kinetics of composite clinical remission. **(A)** Proportion of patients achieving Very Early Response (VER) at 3 months and Early Response (ER) at 6 months. Response was defined as a 30% or greater reduction in proteinuria combined with an eGFR decline of less than 15% from baseline. **(B)** Longitudinal changes in composite clinical remission status over 12 months. The stacked bar chart shows the cumulative proportion of patients achieving complete remission (CR), partial remission (PR), or no remission (NR). CR, complete remission; eGFR, estimated glomerular filtration rate; ER, early response; NR, no remission; PR, partial remission; VER, very early response.

**Figure 6:**
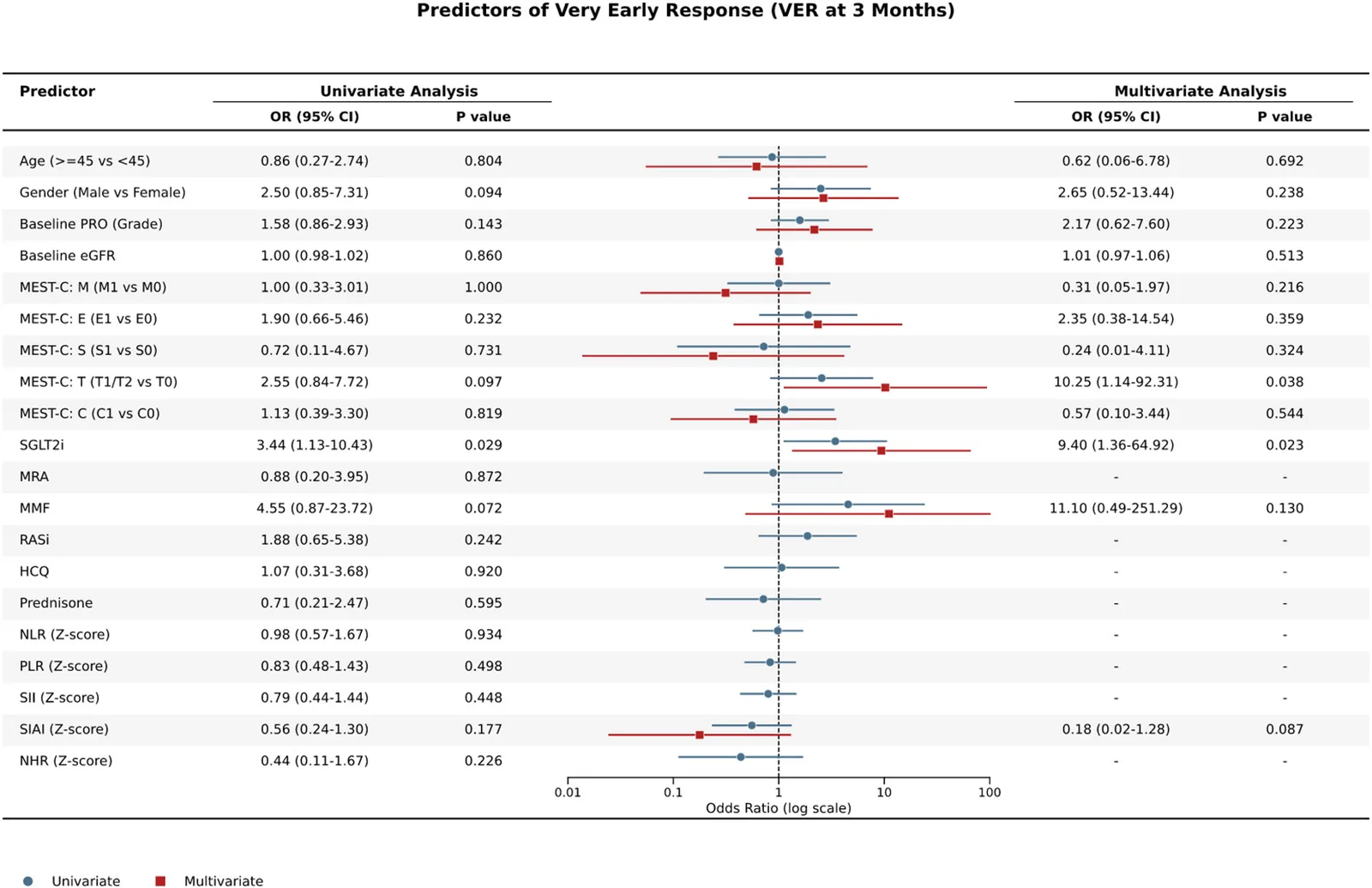
Independent predictors of very early composite clinical responses. Forest plot showing unadjusted (blue circles) and adjusted (red squares) odds ratios with 95% confidence intervals for achieving Very Early Response (VER) at 3 months. The multivariable logistic regression model used an *a priori* covariate inclusion strategy incorporating core clinicopathological variables. Predictors with P values below 0.05 are highlighted in bold. CI, confidence interval; eGFR, estimated glomerular filtration rate; ER, early response; HCQ, hydroxychloroquine; MEST-C, Oxford pathology score; MMF, mycophenolate mofetil; MRA, mineralocorticoid receptor antagonist; OR, odds ratio; PRO, proteinuria; RASi, renin-angiotensin system inhibitors; SGLT2i, sodium-glucose cotransporter-2 inhibitors; VER, very early response.

At 3 months, 52.6% of patients achieved Very Early Response (VER), and 54.4% achieved Early Response (ER) by 6 months (**Figure 5A**). In multivariable logistic regression for VER (**Figure 6**; **Supplementary Table 4**), concomitant use of an SGLT2 inhibitor independently predicted VER (OR 9.40, 95% CI 1.36 to 64.92; P = 0.023). Advanced tubulointerstitial fibrosis (T1/T2) was also positively associated with VER. However, given real-world prescribing patterns in which patients with more severe chronicity often receive intensified combination therapy and closer follow-up, this association likely reflects confounding by indication rather than a direct biological enhancement of TR-budesonide response. Predictors of ER at 6 months were broadly concordant (**Supplementary Figure 1**; **Supplementary Table 8**). Subgroup analysis showed that baseline proteinuria (<1.0 vs. ≥ g/24h) did not significantly modify the 3-month VER rate (42.3% vs. 61.3%, P = 0.189; **Supplementary Table 4**), but patients with higher baseline proteinuria achieved a higher 6-month ER rate (71.0% vs. 38.5%, P = 0.018; **Supplementary Table 8**). In the multivariable Cox model for composite remission, baseline proteinuria strata showed no independent association with long-term remission (P = 0.680, P = 0.940, and P = 0.640, respectively; **Supplementary Table 9**).

### 3.5 Association of endocapillary hypercellularity with accelerated clinical remission kinetics

**Figure 7:**
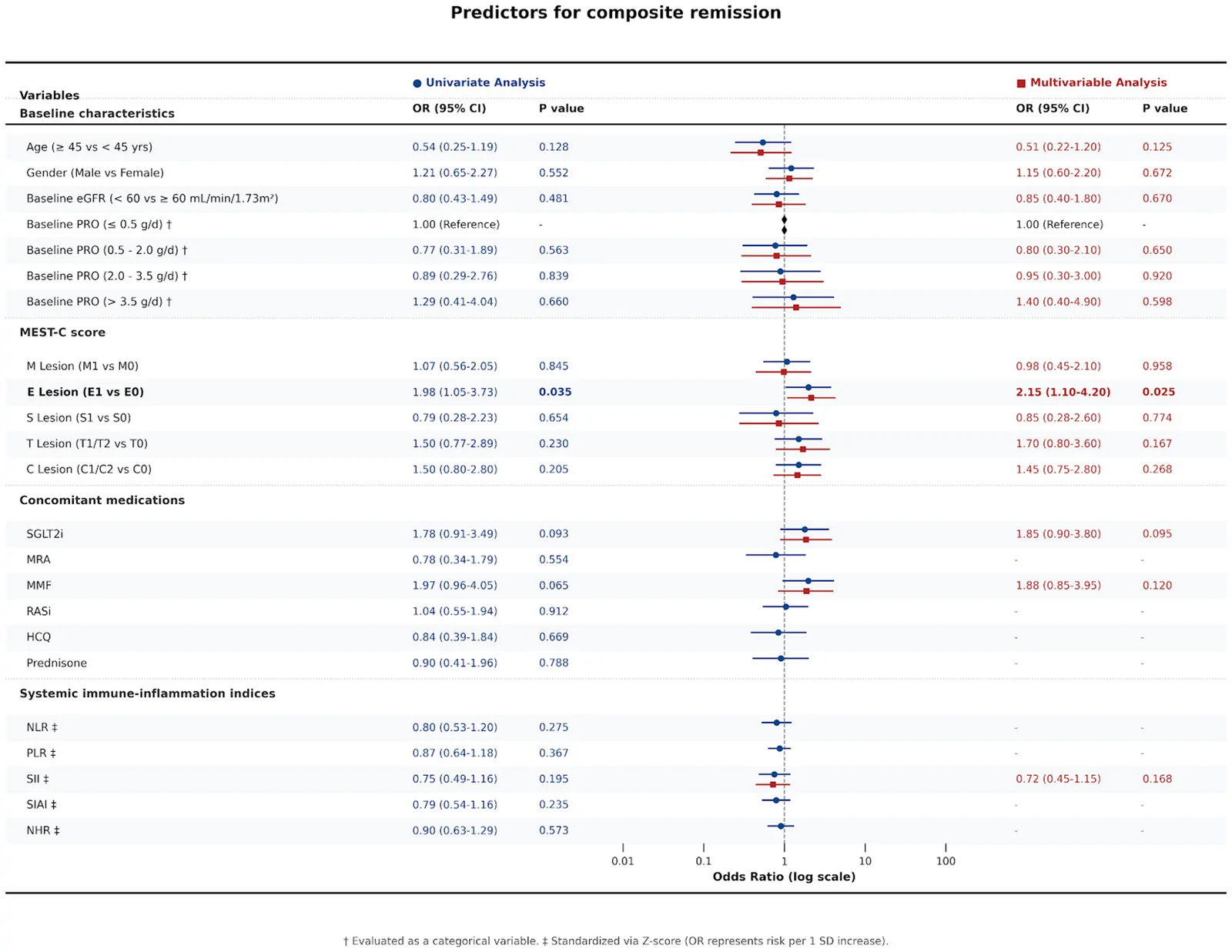
Comprehensive dual-track forest plot of baseline predictors for composite clinical remission (CR/PR). Forest plot showing unadjusted (univariate) and adjusted (multivariable) odds ratios (ORs) with 95% confidence intervals (CIs) for achieving composite clinical remission, defined as complete or partial proteinuria remission with concomitant eGFR stability. Blue circles indicate univariate point estimates, and red squares indicate adjusted multivariable estimates. The x-axis is logarithmic, and the vertical dashed line denotes the null value (OR = 1.0). The multivariable model included systemic immune-inflammation variables with univariate P values below 0.20 (for example, SII), together with prespecified core clinical and pathological covariates and concomitant targeted therapies (SGLT2i and MMF). Variables not retained in the multivariable model are shown as dashes. Continuous immune-inflammation indices were Z-score standardized; ORs therefore represent risk per 1 standard deviation increase. Predictors with P values below 0.05 in the multivariable model are highlighted in bold. CI, confidence interval; OR, odds ratio; eGFR, estimated glomerular filtration rate; PRO, proteinuria; MEST-C, Oxford pathology score; SGLT2i, sodium-glucose cotransporter-2 inhibitors; MMF, mycophenolate mofetil; SII, systemic immune-inflammation index.

**Figure 8:**
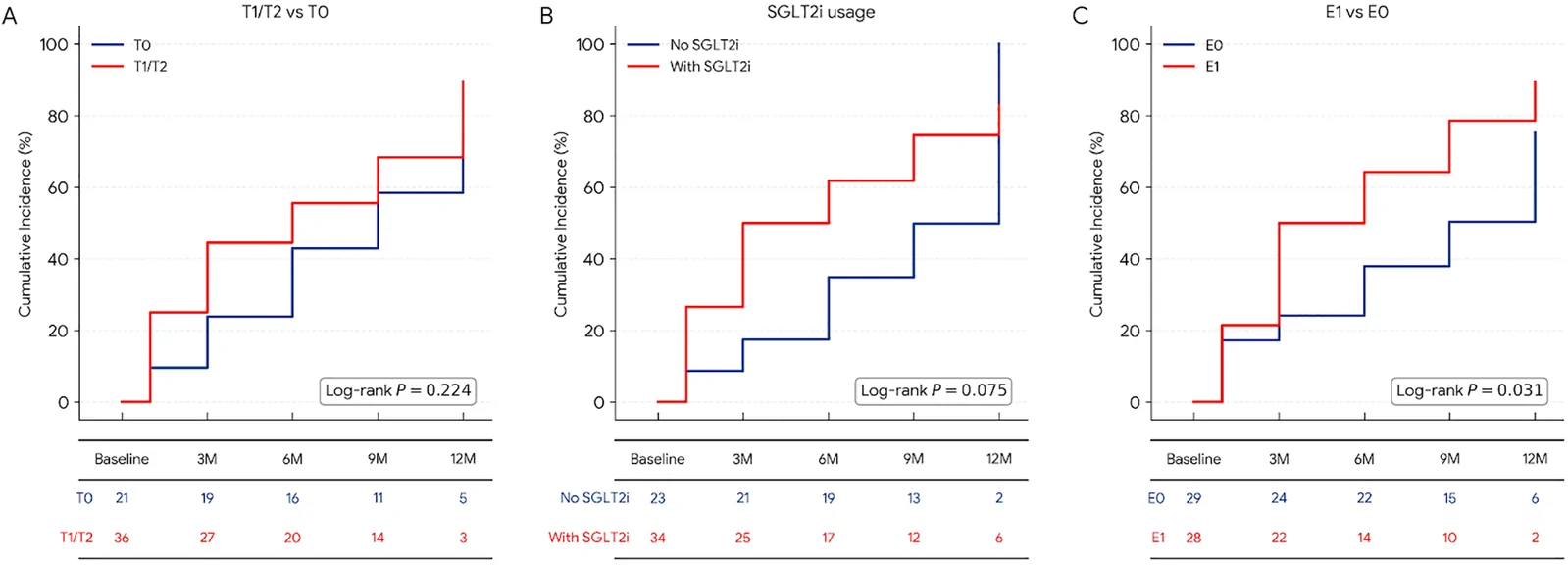
Kaplan-Meier estimates of composite clinical remission stratified by core clinico-pathological determinants. Kaplan-Meier curves depicting time to composite clinical remission (CR/PR). Stratified analyses highlight three prespecified determinants: **(A)** tubulointerstitial atrophy and interstitial fibrosis (T1/T2 vs T0), **(B)** concomitant SGLT2 inhibitor therapy, and **(C)** endocapillary hypercellularity (E1 vs E0). Between-group differences were assessed using the log-rank test (panel C log-rank P = 0.031). CR, complete remission; PR, partial remission; SGLT2i, sodium-glucose cotransporter-2 inhibitors.

Over 12 months, the proportion of patients achieving partial or complete remission increased progressively (**Figure 5B**). In models evaluating predictors of overall composite clinical remission (**Figure 7**), the associations observed for SGLT2 inhibitor use and T lesions at earlier milestones were attenuated (**Supplementary Table 9**). In contrast, baseline endocapillary hypercellularity (E1) emerged as the only independent predictor of composite remission (HR 2.20; P = 0.022). Kaplan–Meier analyses supported these findings (**Figure 8**). Remission curves stratified by tubulointerstitial fibrosis demonstrated similar overall remission probabilities despite differences in baseline chronicity (**Figure 8A**). Stratification by concomitant SGLT2 inhibitor therapy showed earlier remission among treated patients, consistent with an accelerated response profile (**Figure 8B**). Most notably, patients with E1 lesions achieved remission more rapidly than those with E0 lesions (**Figure 8C**; log-rank P = 0.031), supporting E1 as a marker of a treatment-responsive inflammatory phenotype. No other baseline demographic or clinical factors significantly differentiated time to remission (**Supplementary Figures 2**, **S3**).

To address potential confounding by disease duration, we evaluated the interval between renal biopsy and TR-budesonide initiation (median 23.27 months [IQR: 1.50–79.77]). Patients with the active E1 phenotype started therapy much sooner after their biopsy than those with E0 lesions (median 6.63 months [IQR: 0.28–53.62] vs. 33.93 months [IQR: 15.17–93.47]; P = 0.055), reflecting a clinical preference for treating highly active inflammatory states early. To determine whether this prognostic relationship depended on timing, a multivariable interaction test was performed at the 6-month milestone (N = 57). The interaction term between E1 status and the biopsy-to-treatment interval was non-significant (β = -0.0129, P_interaction_ = 0.222; **Supplementary Table 10**), indicating that treatment timing did not statistically modify the prognostic value of E1 across the broader cohort, though this exploratory assessment was inherently constrained by statistical power.

To further evaluate this temporal dynamic within an immediate therapeutic window, a sensitivity analysis was restricted to the high-proximity subgroup of 16 patients who initiated therapy within 3.0 months post-biopsy. Within this subset, patients with E1 lesions exhibited a higher 6-month early response rate than those with E0 lesions (66.7% [8/12] vs. 25.0% [1/4]). However, while this numerical elevation yielded a large odds ratio (β = 1.792, OR = 6.00, P = 0.170; **Supplementary Table 11**), it did not reach statistical significance owing to the small sample size. Consequently, while this exploratory observation points toward a plausible directional trend of enhanced early response within an immediate window, it remains statistically inconclusive and requires validation in larger cohorts.

### 3.6 Development and internal validation of predictive nomograms

**Figure 9:**
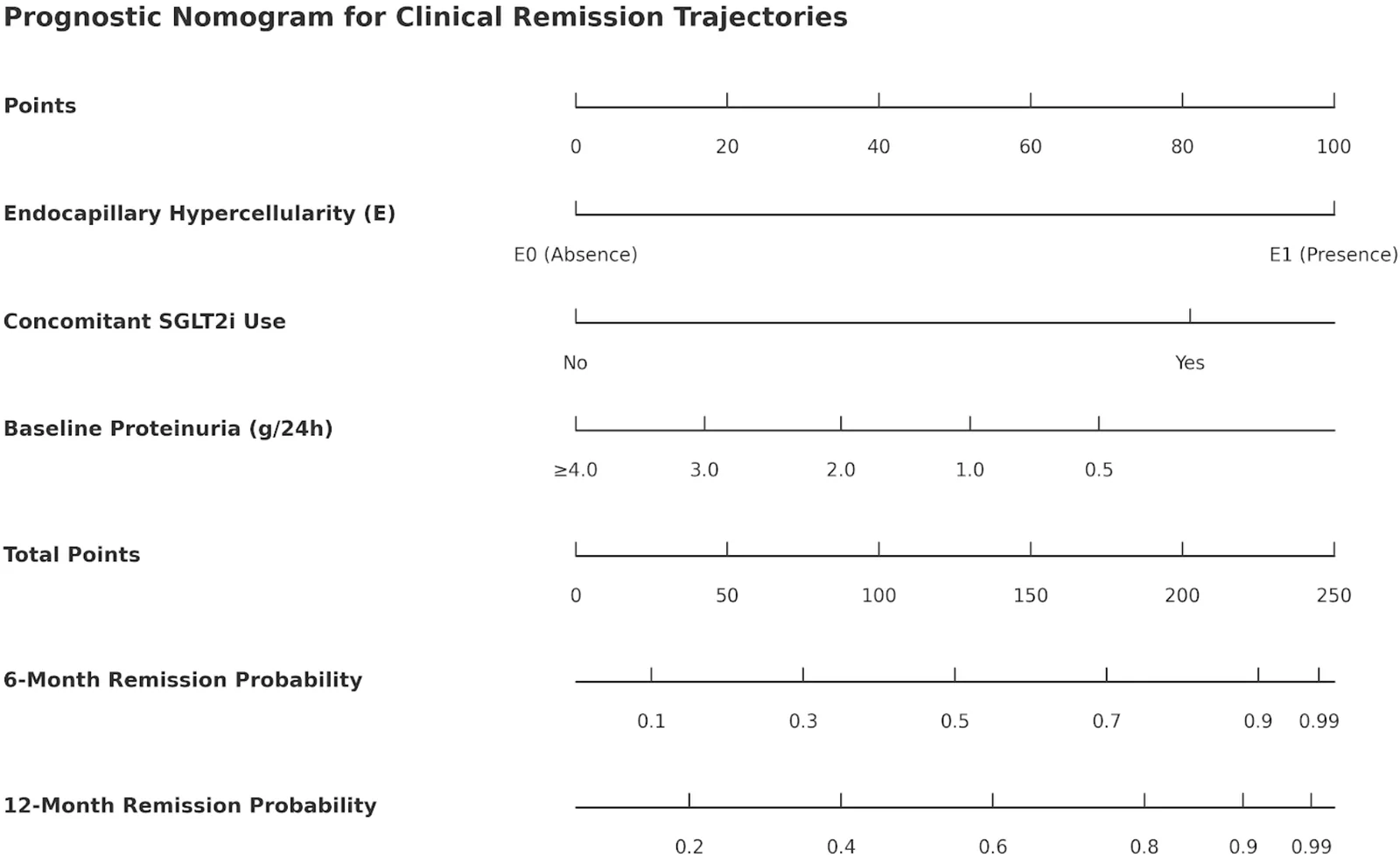
Prognostic Nomogram for Predicting Composite Clinical Remission (CR/PR). The nomogram integrates key baseline clinical variables, including Endocapillary Hypercellularity (E), concomitant SGLT2i use, and baseline proteinuria, to estimate individualized probabilities of achieving composite clinical remission at 6 and 12 months. Composite remission is defined as complete remission (proteinuria below 0.3 g/day) or partial remission (50% or greater proteinuria reduction with an absolute value below 3.5 g/day), together with eGFR stability (decline less than 15% from baseline). Individual scores are assigned for each variable, summed to a total score, and mapped to the corresponding remission probability at each time point. CR, complete remission; PR, partial remission; SGLT2i, sodium-glucose cotransporter-2 inhibitors.

**Figure 10:**
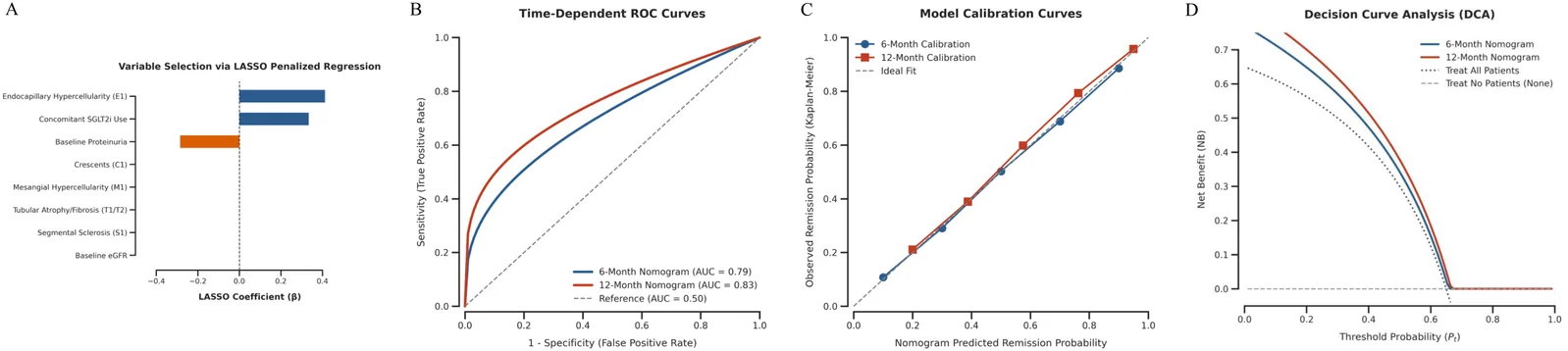
Performance and exploratory Clinical applicability of the proposed CR/PR Prognostic Nomogram. **(A)** LASSO-penalized Cox regression feature selection identifying key baseline clinical and histological predictors. **(B)** Time-dependent receiver operating characteristic (ROC) curves demonstrating discrimination with an AUC of 0.79 at 6 months and 0.83 at 12 months. **(C)** Calibration plots comparing nomogram-predicted remission probabilities with actual observed estimates; alignment with the 45-degree reference line indicates acceptable model calibration. **(D)** Decision curve analysis (DCA) illustrating favorable net clinical benefit across a wide range of threshold probabilities compared to treat-all or treat-none strategies, supporting the model’s potential exploratory utility. AUC, area under the curve; DCA, decision curve analysis; ROC, receiver operating characteristic.

To complement our primary multivariable models and guard against over-optimism, variable selection was streamlined using a LASSO-penalized Cox regression workflow with 10-fold cross-validation (**Supplementary Table 12**). This identified baseline proteinuria, E1 status, and SGLT2i use as core robust predictors, whose coefficients and point allocations directly shaped the prognostic nomogram (**Figure 9**). The model demonstrated stable discrimination, through LASSO-based feature selection (**Figure 10A**), yielding time-dependent AUC values of 0.79 at 6 months and 0.83 at 12 months (**Figure 10B**). Calibration curves showed acceptable agreement between the predicted probabilities and observed estimates (**Figure 10C**), while decision curve analysis (DCA) demonstrated a positive net clinical benefit across relevant threshold probabilities, supporting its exploratory utility for clinical decision-making (**Figure 10D**). To isolate the potential confounding influence of treatment timing and early SGLT2i-associated hemodynamic effects, patients were stratified into non-users (n = 23), pre-existing stable users (n = 15), and concurrent or new users (n = 19). At 3 months, the unadjusted very early response (VER) rate was 34.8% in non-users, 60.0% in pre-existing users, and 68.4% in concurrent/new users (**Supplementary Table 9**). In the multivariable logistic regression model adjusted for Oxford lesions and baseline clinical severity, this time-stratified analysis revealed that concurrent new initiation of SGLT2i trended strongly toward predicting 3-month VER (Adjusted OR = 3.805, 95%CI: 0.967-14.981, P = 0.056), whereas pre-existing stable use showed a weaker association (Adjusted OR = 3.257, 95%CI: 0.793-13.380, P = 0.102; **Supplementary Table 13**). These findings suggest that concomitant SGLT2i use is associated with favorable early proteinuric trajectories in this real-world cohort, an effect likely mediated by immediate hemodynamic actions.

## 4 Discussion

This study extends evidence beyond clinical trial endpoints by characterizing the real-world response kinetics of targeted-release (TR) budesonide across the heterogeneous Oxford MEST-C spectrum. Although the phase 3 NefIgArd trial established the efficacy of TR-budesonide in reducing proteinuria and preserving kidney function (10, 11), clinical practice raises additional questions regarding how baseline pathology and concomitant therapies shape the timing and depth of remission. In this cohort, TR-budesonide produced consistent proportional reductions in proteinuria across histopathological strata, including patients with substantial tubulointerstitial fibrosis. Early response was strongly associated with concomitant SGLT2 inhibitor therapy, whereas long-term composite remission was most closely linked to the presence of endocapillary hypercellularity (E1). A central finding is that E1 lesions were associated with faster clinical remission kinetics within this treated cohort. Endocapillary hypercellularity is traditionally considered a marker of active, progressive disease that may respond to systemic corticosteroids, albeit with considerable systemic toxicity (14, 16). Our results suggest that the E1 lesion may identify a biologically active inflammatory state that features higher intrinsic reversibility. Mechanistically, E1 reflects active mesangial-endothelial injury, likely sustained by ongoing deposition of mucosal-derived galactose-deficient IgA1 (Gd-IgA1) containing immune complexes and subsequent local inflammatory signaling (2, 16). While TR-budesonide is designed to intercept pathogenic Gd-IgA1 production via upstream intestinal mucosal suppression (Hit 1) (3, 9), our study did not measure systemic or mucosal immunological biomarkers (e.g., Gd-IgA1 or immune complexes). Consequently, our findings primarily describe clinical remission trajectories and should not be interpreted as direct mechanistic evidence of mucosal immune suppression. While E1 appears to be a practical marker for a treatment-responsive inflammatory window, controlled trials are needed to clarify whether this responsiveness stems from upstream mucosal intervention or inherent tissue reversibility. Equally important, TR-budesonide appeared to retain antiproteinuric efficacy in the setting of established chronic injury. Advanced tubulointerstitial fibrosis (T lesions) and segmental glomerulosclerosis (S lesions) often indicate irreversible structural damage and are commonly associated with reduced benefit and higher risk from conventional systemic immunosuppression (15, 27). In contrast, we observed comparable proportional proteinuria reductions across MEST-C strata, including patients with T1/T2 lesions. One explanation is that TR-budesonide primarily acts at the distal ileum and undergoes extensive first-pass hepatic metabolism (8), thereby targeting the upstream immune source without requiring high systemic exposure or direct drug delivery to chronically scarred renal tissue. Crucially, these findings must be interpreted as exploratory observations rather than definitive evidence of unattenuated drug efficacy. Because segmental glomerulosclerosis (S1) was present in 91.2% of our cohort, the sparse S0 reference group precludes robust comparative conclusions regarding this lesion. Furthermore, since these real-world trajectories occurred in the context of comprehensive background therapies, including maximally tolerated RAS blockade and SGLT2 inhibitors, the parallel proteinuria reductions in chronic phenotypes reflect the efficacy of an integrated multi-drug regimen rather than the isolated effect of TR-budesonide. As a result, its upstream mechanism may remain effective even when downstream renal fibrosis is advanced. The predictors of very early response (VER) require careful interpretation. Although T1/T2 lesions were associated with achieving VER, this association is unlikely to reflect a direct biological potentiation by fibrosis. In routine practice, patients with more advanced chronicity and higher risk features often receive intensified background therapy and closer follow-up, including maximally tolerated RAS blockade and SGLT2 inhibitors. Therefore, this finding is more consistent with confounding by indication, in which comprehensive standard-of-care therapy contributes to an early reduction in proteinuria. Nonetheless, demonstrating proportional proteinuria improvement in high-chronicity phenotypes remains clinically meaningful because proteinuria reduction is strongly linked to improved long-term renal outcomes (20).

Our findings also support a mechanistic rationale for combined upstream and downstream intervention. Concomitant SGLT2 inhibitor therapy was associated with a markedly higher likelihood of achieving early milestones, consistent with rapid hemodynamic effects that lower intraglomerular pressure and reduce proteinuria (7, 28). SGLT2 inhibitors also mitigate downstream hypoxia and tubulointerstitial stress, which may influence progression pathways that are less directly addressed by gut-targeted immunomodulation (hit 4) (29). In contrast, TR-budesonide is expected to act more gradually by suppressing mucosal immune activation and reducing pathogenic Gd-IgA1 production. Together, these complementary mechanisms provide a coherent explanation for an early acceleration of response with SGLT2 inhibitors and a longer-term trajectory toward deeper remission that is most apparent in patients with active inflammatory lesions such as E1. To facilitate translation into clinical decision-making, we developed and internally validated a time-dependent Cox nomogram integrating MEST-C pathology, baseline clinical parameters, and concomitant therapies. Existing risk tools estimate the natural history of IgAN under standard care (30), whereas our model was designed to individualize remission expectations specifically in patients treated with TR-budesonide. This therapy-focused approach may support more informed counseling, individualized monitoring strategies, and shared decision-making regarding combination therapy. Several limitations should be considered. The retrospective, single-center design limits causal inference and reduces power for detailed subgroup analyses. In addition, the observation window reflects early real-world implementation of a newly adopted therapy. Accordingly, we prioritized categorical eGFR stability and remission kinetics rather than short-term eGFR slope, which can be distorted by early, reversible hemodynamic changes after SGLT2 inhibitor or RAS inhibitor initiation (21, 22). Longer follow-up and external validation in multicenter cohorts are needed to confirm model transportability and to evaluate associations with hard renal endpoints. Furthermore, the retrospective, single-center design and the inclusion of a homogeneous Chinese population restrict the direct generalizability of our findings to multi-ethnic or Western populations. The lack of an untreated or standard-of-care control group prevents us from separating the true drug-specific predictive re sponses from inherent prognostic components. Additionally, these exploratory analyses were evaluated at nominal significance levels without multiplicity correction; thus, their P values should be viewed with caution regarding potential type I error. Finally, since our prognostic nomogram was built upon a relatively small event-to-variable ratio, it must be interpreted strictly as an exploratory, hypothesis-generating tool that mandates robust external validation before being considered for clinical implementation.

## 5 Conclusions

Targeted-release budesonide was associated with consistent proportional reductions in proteinuria across diverse Oxford MEST-C phenotypes, including in patients presenting with substantial tubulointerstitial fibrosis. Early response was strongly associated with concomitant SGLT2 inhibitor therapy, while histological E1 lesions identified a treatment-responsive inflammatory phenotype characterized by accelerated remission kinetics. Although a prognostic nomogram integrating pathology, baseline clinical factors, and concurrent therapies provides a practical framework to individualize remission expectations, these real-world findings should be interpreted as exploratory and hypothesis-generating. Further validation in larger, controlled, multi-center cohorts is essential before broader clinical implementation.

## Data Availability

The raw data supporting the conclusions of this article will be made available by the authors, without undue reservation.
