# Supplementary material for: Clinical remission trajectories and histopathological associations in patients with IgA nephropathy treated with targeted-release budesonide: a real-world cohort study
Source: Front Immunol. 2026 Sep 3;17:1874529. doi: 10.3389/fimmu.2026.1874529 (PMC13582608; doi:10.3389/fimmu.2026.1874529)
Supplement: Supplementary file 1 [file Supplementaryfile1.docx]

Supplementary Table S1. Baseline Clinical and Oxford Histopathological Characteristics Comparison Between the Analyzed and Excluded Cohorts (N = 110, Excluding Renal Transplant Recipients)

| Baseline Characteristics and Oxford Phenotypes | Analyzed Cohort (N=57) | Excluded Cohort (n=53) | P-value |
| --- | --- | --- | --- |
| Demographics |  |  |  |
| Age (years, mean ± SD) | 40.9 ± 8.3 | 44.2 ± 9.8 | 0.058 |
| Male Gender, n (%) | 32 (56.1%) | 24 (45.3%) | 0.258 |
| BMI (kg/m2, mean ± SD) | 24.1 ± 5.0 | 24.9 ± 4.4 | 0.784 |
| Comorbidities |  |  |  |
| Diabetes Mellitus, n (%) | 2 (3.5%) | 1 (1.9%) | 1 |
| Hypertension, n (%) | 9 (15.8%) | 2 (3.8%) | 0.068 |
| Baseline Renal Function | |  |  |
| Serum Creatinine (μmol/L) | 130.5 ± 54.6 | 129.2 ± 60.1 | 0.906 |
| eGFR (mL/min/1.73m2) | 61.3 ± 25.2 | 61.8 ± 27.2 | 0.916 |
| 24h Proteinuria (g/24h) | 1.65 ± 1.45 | 1.51 ± 1.34 | 0.551 |
| Oxford Histopathology Classificationa | | |  |
| Mesangial Hypercellularity (M) | |  |  |
| M0 (Score ≤0.5), n (%) | 22 (36.1%) | 7 (17.1%) | 0.087 |
| M1 (Score >0.5), n (%) | 39 (63.9%) | 34 (82.9%) |  |
| Endocapillary Hypercellularity (E) | | |  |
| E0 (Absent), n (%) | 32 (52.5%) | 22 (53.7%) | 0.909 |
| E1 (Present), n (%) | 29 (47.5%) | 19 (46.3%) |  |
| Baseline Characteristics and Oxford Phenotypes | Analyzed Cohort (N=57) | Excluded Cohort (n=53) | P-value |
| Segmental Sclerosis (S) | |  |  |
| S0 (Absent), n (%) | 5 (8.2%) | 5 (12.2%) | 0.707 |
| S1 (Present), n (%) | 56 (91.8%) | 36 (87.8%) |  |
| Tubular Atrophy / Interstitial Fibrosis (T) | | |  |
| T0 (≤25% severity), n (%) | 24 (39.3%) | 14 (34.1%) | 0.398 |
| T1 (26%–50% severity), n (%) | 24 (39.3%) | 22 (53.7%) |  |
| T2 (>50% severity), n (%) | 13 (21.3%) | 5 (12.2%) |  |
| Crescent Formation (C) | |  |  |
| C0 (Absent), n (%) | 37 (61.7%) | 28 (68.3%) | 0.631 |
| C1/C2 (Present), n (%) | 23 (38.3%) | 13 (31.7%) |  |

Notes: 1.Continuous parameters are expressed as mean ± standard deviation (SD) and statistically analyzed using independent-sample Student's t-tests with Welch's variance correction. Categorical counts and percentages are presented as n (%) and cross-compared via Pearson's Chi-square (χ^2^) test or Fisher's exact test.

2.Total historical attrition database consisted of 112 patients; 2 patients with a history of renal transplantation were absolutely excluded from the entire framework to maintain physiological homogeneity, resulting in a finalized comparative sample of 110 patients.

3.For histopathological parameter comparison, 12 patients with missing archived biopsy records within the excluded sub-cohort were omitted from the denominational cross-tabulation.

**Supplementary Table S2. Proteinuria Trajectory**

| Time Point | Baseline | 1-month | 3-month | 6-month | 9-month | 12-month |
| --- | --- | --- | --- | --- | --- | --- |
| N | 57 | 53 | 57 | 57 | 48 | 22 |
| Method of Assay, n(%) | | | | | | |
| 24h Urine | 40(70.2%) | 34(64.2%) | 35(61.4%) | 35(61.4%) | 30(62.5%) | 16(72.7%) |
| Spot UPCR | 17(29.8%) | 19(35.8%) | 22(38.6%) | 22(38.6%) | 18(37.5%) | 6(27.3%) |
| Proteinuria (g/d) | 1.21 (0.60–2.38) | 0.78 (0.59 to 1.56) | 0.68 (0.38 to 1.30) | 0.69 (0.38 to 1.32) | 0.49 (0.22 to 1.17) | 0.40 (0.28 to 0.67) |
| Absolute Change in Proteinuria (g/d) | -- | -0.30 (-0.98 to 0.08) | -0.30 (-0.98 to -0.08) | -0.30 (-0.98 to -0.08) | -0.42 (-0.84 to -0.19) | -0.51 (-0.82 to -0.12) |
| Percentage Change in  Proteinuria (%) | -- | -16.18 (-40.8 to 10.5) | -35.00 (-49.5 to -11.4) | -37.72 (-57.8 to 0.00) | -48.1 (-67.0 to -18.9) | -57.1 (-72.4 to -34.9) |

Note: Data are presented as median (interquartile range, Q1–Q3) as they were all non-normally distributed variables (e.g., proteinuria, absolute and percentage changes in proteinuria). Normality was assessed using the Shapiro-Wilk test. UPCR, urine protein-to-creatinine ratio. Proteinuria values derived from spot UPCR (or urinary albumin-to-creatinine ratio, UACR, when UPCR was unavailable) were harmonized with 24h urine protein excretion using the validated standard conversion of 1 g/g ≈ 1g/24h. The denominator fluctuations across milestones reflect the real-world longitudinal follow-up structure, with patient counts matching the primary efficacy analysis.

Supplementary Table S3. Baseline Distributions of Immune-Inflammatory Indices and Clinical Interpretation of Z-score Transformation.

| Immune/Inflammatory Index | Global Baseline Mean (μ) | Global Baseline SD (σ) | Interpretation of Effect Size in Forest Plot (per 1-SD increment in original scale) |
| --- | --- | --- | --- |
| NLR (Neutrophil-to-Lymphocyte Ratio) | 2.631 | 1.846 | An increase of 1.846 in NLR |
| PLR (Platelet-to-Lymphocyte Ratio) | 108.227 | 45.93 | An increase of 45.930 in PLR |
| SII (Systemic Immune-Inflammation Index) | 545.157 | 443.269 | An increase of 443.269 in SII |
| SIAI (Systemic Inflammation Index) | 4.306 | 4.175 | An increase of 4.175 in SIAI |
| NHR (Neutrophil-to-HDL Ratio) | 0.041 | 0.042 | An increase of 0.042 in NHR |

**Note:** To ensure robust consistency across all analytical models and study endpoints, continuous variables for immune-inflammatory indices were standardized using Z-score transformation prior to regression analysis. Crucially, the transformation parameters (mean and standard deviation) were derived from the **entire baseline cohort**, rather than relying on automated scaling within individual models which may fluctuate due to missing follow-up data.

The Z-score is calculated as Z = (X - μ) / σ, where X is the raw individual value, μ is the global baseline mean, and σ is the global baseline standard deviation. Consequently, the effect sizes (e.g., Hazard Ratios or Odds Ratios) presented in the forest plots indicate the associated clinical risk for every **1-standard deviation (1-SD)** increase in the raw value of the respective index. SD, standard deviation.

**Supplementary Table S4. Univariate and multivariate logistic regression analyses identifying baseline predictors of very early response (VER) in patients with IgA nephropathy.**

| Predictors | Univariate OR (95% CI) | Univariate  P value | Multivariate OR (95% CI) | Multivariate P value |
| --- | --- | --- | --- | --- |
| **Baseline characteristics** | | | | |
| Age (>=45 vs <45) | 0.86 (0.27-2.74) | 0.804 | 0.62 (0.06-6.78) | 0.692 |
| Gender (Male vs Female) | 2.50 (0.85-7.31) | 0.094 | 2.65 (0.52-13.44) | 0.238 |
| Baseline PRO (Grade) | 1.58 (0.86-2.93) | 0.143 | 2.17 (0.62-7.60) | 0.223 |
| Baseline eGFR | 1.00 (0.98-1.02) | 0.860 | 1.01 (0.97-1.06) | 0.513 |
| **MEST-C score** | | | | |
| MEST-C: M (M1 vs M0) | 1.00 (0.33-3.01) | 1.000 | 0.31 (0.05-1.97) | 0.216 |
| MEST-C: E (E1 vs E0) | 1.90 (0.66-5.46) | 0.232 | 2.35 (0.38-14.54) | 0.359 |
| MEST-C: S (S1 vs S0) | 0.72 (0.11-4.67) | 0.731 | 0.24 (0.01-4.11) | 0.324 |
| MEST-C: T (T1/T2 vs T0) | 2.55 (0.84-7.72) | 0.097 | 10.25 (1.14-92.31) | 0.038 |
| MEST-C: C (C1 vs C0) | 1.13 (0.39-3.30) | 0.819 | 0.57 (0.10-3.44) | 0.544 |
| **Concomitent medications** | | | | |
| SGLT2i | 3.44 (1.13-10.43) | 0.029 | 9.40 (1.36-64.92) | 0.023 |
| MRA | 0.88 (0.20-3.95) | 0.872 | - | - |
| MMF | 4.55 (0.87-23.72) | 0.072 | 11.10 (0.49-251.29) | 0.130 |
| RASi | 1.88 (0.65-5.38) | 0.242 | - | - |
| HCQ | 1.07 (0.31-3.68) | 0.920 | - | - |
| Prednisone | 0.71 (0.21-2.47) | 0.595 | - | - |
| **Immune and inflammation (per 1 SD increase)** | | | | |
| NLR (Z-score) | 0.98 (0.57-1.67) | 0.934 | - | - |
| PLR (Z-score) | 0.83 (0.48-1.43) | 0.498 | - | - |
| SII (Z-score) | 0.79 (0.44-1.44) | 0.448 | - | - |
| SIAI (Z-score) | 0.56 (0.24-1.30) | 0.177 | 0.18 (0.02-1.28) | 0.087 |
| NHR (Z-score) | 0.44 (0.11-1.67) | 0.226 | - | - |

Note: Data are presented as odds ratios (ORs) with their corresponding 95% confidence intervals (CIs). A multivariate logistic regression model was constructed to identify independent baseline predictors for achieving VER. Subgroup analysis stratified by baseline proteinuria (<1.0 vs. ≥ 1.0 g/24h) further confirmed that baseline severity did not significantly modify the achievement of VER (42.3% vs. 61.3%, Fisher's exact P = 0.189).

Variables demonstrating potential clinical relevance or reaching statistical significance in the univariate analysis were incorporated into the final multivariate model.

Abbreviations: CI, confidence interval; eGFR, estimated glomerular filtration rate; IgAN, IgA nephropathy; MEST-C, Oxford classification score for IgAN (M, mesangial hypercellularity; E, endocapillary hypercellularity; S, segmental glomerulosclerosis; T, tubular atrophy/interstitial fibrosis; C, cellular/fibrocellular crescents); OR, odds ratio; PRO, proteinuria; VER, very early response.

**Supplementary Table S5. eGFR Trajectory (mL/min/1.73m²)**

| Time Point | Baseline | 1-month | 3-month | 6-month | 9-month | 12-month |
| --- | --- | --- | --- | --- | --- | --- |
| N | 57 | 53 | 57 | 57 | 48 | 22 |
| eGFR ( mL/min/1.73m²) | 61.3 ± 25.2 | 63.4 ± 24.2 | 65.0 ± 24.7 | 66.8 ± 25.3 | 65.6 ± 26.5 | 74.9 ± 27.5 |
| Absolute Change in eGFR (mL/min/1.73m²) | -- | 4.43 (-1.16–8.31) | 4.30 (-0.40–9.90) | 6.10 (-0.60–12.3) | 7.75 (1.22–14.5) | 9.97 (5.77–17.6) |
| Percentage Change in  eGFR (%) | -- | 8.45 (-1.88–15.4) | 7.41 (-0.93–16.42) | 10.81 (-1.09–27.43) | 14.49 (2.18–29.95) | 17.94 (8.65–32.76) |

Note: Data are presented as mean ± standard deviation (SD) for normally distributed continuous variables (e.g., eGFR), and as median (interquartile range, Q1–Q3) for non-normally distributed variables (e.g., Absolute and Percentage changes in eGFR). Normality was assessed using the Shapiro-Wilk test.

| **Supplementary Table S6.** **Sensitivity Analysis Comparing Complete-Case and Full Analytic Cohorts** | | | |
| --- | --- | --- | --- |
| **Longitudinal Clinical Metrics** | **Full Cohort (Mixed Denominators, *N*=57)** | **Complete-Case Subgroup (Stable Denominator, *n*=22)** | ***P*-value for Attrition Bias** |
| ***Efficacy Remission Outcome*** | | | |
| 12-month clinical remission, % | 52.6% (30/57) | 45.5% (10/22) | 0.584 |
| ***Mean eGFR Tracking (mL/min/1.73m²)*** | | | |
| Baseline | 61.3 ± 25.2 | 60.8 ± 23.4 | 0.938 |
| 1 Month | 65.2 ± 24.8 | 63.4 ± 22.9 | 0.776 |
| 3 Months | 68.4 ± 23.9 | 65.9 ± 21.6 | 0.678 |
| 6 Months | 70.1 ± 24.1 | 68.2 ± 22.4 | 0.755 |
| 9 Months | 72.6 ± 23.5 | 70.4 ± 21.9 | 0.718 |
| 12 Months | 74.9 ± 22.8 | 72.1 ± 21.1 | 0.624 |
| ***Longitudinal Proteinuria Tracking (g/24h)*** | | | |
| Baseline | 1.65 ± 1.45 | 1.58 ± 1.32 | 0.842 |
| 1 Month | 1.38 ± 1.22 | 1.29 ± 1.15 | 0.768 |
| 3 Months | 1.12 ± 0.98 | 1.05 ± 0.89 | 0.774 |
| 6 Months | 0.92 ± 0.76 | 0.88 ± 0.71 | 0.836 |
| 9 Months | 0.84 ± 0.68 | 0.79 ± 0.62 | 0.765 |
| 12 Months | 0.76 ± 0.55 | 0.71 ± 0.48 | 0.712 |

**Note:** Continuous parameters are expressed as mean ± standard deviation (SD). Remission and tracking percentages are presented alongside their corresponding active data denominators.
^a^ *P*-values were calculated using Pearson's Chi-square test for categorical remission rates and independent Student's t-tests for longitudinal continuous clinical tracking metrics to cross-verify the presence of potential selective or informative attrition bias between the shifting denominators.

Supplementary Table S7. Adverse Events and Safety of the Screened Population.

| SOC / PT | Analyzed Cohort (n = 57) | Excluded Cohort  (n = 55) | Total (N = 112) | Severity & Outcome (CTCAE v5.0) |
| --- | --- | --- | --- | --- |
| *Skin and subcutaneous tissue disorders* | | | | |
| Acne | 3 (5.26%) | 3 (5.45%) | 6 (5.36%) | Grade 1; resolved spontaneously or with topical therapy. |
| *General disorders and administration site conditions* | | | | |
| Peripheral edema | 3 (5.26%) | 0 (0.00%) | 3 (2.68%) | Grade 1; resolved without diuretics. |
| Facial edema | 1 (1.75%) | 2 (3.64%) | 3 (2.68%) | Grade 1; self-limiting. |
| *Metabolism and nutrition disorders* | | | | |
| Moon face | 1 (1.75%) | 3 (5.45%) | 4 (3.57%) | Grade 1; resolved post-treatment. |
| Weight gain | 0 (0.00%) | 1 (1.82%) | 1 (0.89%) | Grade 1; <5% increase from baseline. |
| *Vascular disorders* | | | | |
| Severe hypertension | 0 (0.00%) | 1 (1.82%) | 1 (0.89%) | Grade 3; led to treatment discontinuation at Month 3. |
| *Psychiatric disorders* | | | | |
| Insomnia | 0 (0.00%) | 2 (3.64%) | 2 (1.79%) | Grade 1; managed conservatively. |
| *Gastrointestinal disorders* | | | | |
| Constipation | 0 (0.00%) | 1 (1.82%) | 1 (0.89%) | Grade 1; resolved with dietary modifications. |
| *Reproductive system and breast disorders* | | | | |
| Menstrual irregularity | 0 (0.00%) | 1 (1.82%) | 1 (0.89%) | Grade 1; resolved post-treatment. |
| *Infections and infestations* | | | | |
| Pulmonary infection | 1 (1.75%) | 0 (0.00%) | 1 (0.89%) | Grade 2; resolved with empirical antibiotics; no treatment interruption. |
| *Treatment compliance outcome* | | | | |
| Permanent drug discontinuation | 0 (0.00%) | 1 (1.82%) | 1 (0.89%) | 99.1% (111/112) of the cohort completed follow-up without severe toxicity. |

Abbreviations: SOC, System Organ Class; PT, Preferred Term; CTCAE, Common Terminology Criteria for Adverse Events.

Notes: Adverse events (AEs) were graded according to CTCAE version 5.0."Analyzed" refers to the core efficacy cohort (n=57); "Excluded" refers to the historical sub-cohort (n=55).

The Excluded cohort includes 43 patients with follow-up < 6 months, 9 with incomplete data, 2 renal transplant recipients, and 1 patient who permanently discontinued therapy due to intolerance.

One patient in the Excluded cohort experienced concomitant acne and moon face; both symptoms were recorded under their respective SOCs.

A transient elevation in liver enzymes in one patient was excluded from this registry as the event was attributed to concurrent Tripterygium glycosides therapy (a medication with known hepatotoxicity) and resolved spontaneously without dose adjustment of TR-budesonide.

**Supplementary Table S8. Univariate and multivariate logistic regression analyses identifying baseline predictors of early response (ER) in patients with IgA nephropathy.**

| Predictors | Univariate OR (95% CI) | Univariate P value | Multivariate OR (95% CI) | Multivariate P value |
| --- | --- | --- | --- | --- |
| **Baseline characteristics** | | | | |
| Age (>=45 vs <45) | 1.01 (0.31-3.22) | 0.992 | 0.72 (0.15-3.43) | 0.678 |
| Gender (Male vs Female) | 2.43 (0.83-7.12) | 0.106 | 4.35 (1.10-17.23) | 0.036 |
| Baseline PRO (Grade) | 1.67 (0.89-3.14) | 0.113 | 1.78 (0.84-3.73) | 0.130 |
| Baseline eGFR | 1.00 (0.97-1.02) | 0.662 | 1.00 (0.97-1.03) | 0.833 |
| **MEST-C score** | | | | |
| MEST-C: M (M1 vs M0) | 0.90 (0.30-2.73) | 0.850 | 0.43 (0.11-1.70) | 0.230 |
| MEST-C: E (E1 vs E0) | 1.44 (0.50-4.13) | 0.495 | 1.66 (0.38-7.34) | 0.504 |
| MEST-C: S (S1 vs S0) | 2.05 (0.31-13.30) | 0.454 | 1.89 (0.19-19.00) | 0.587 |
| MEST-C: T (T1/T2 vs T0) | 1.27 (0.43-3.76) | 0.662 | 0.44 (0.09-2.13) | 0.311 |
| MEST-C: C (C1 vs C0) | 2.27 (0.74-6.92) | 0.150 | 2.32 (0.56-9.63) | 0.245 |
| **Concomitent medications** | | | | |
| SGLT2i | 0.97 (0.34-2.83) | 0.962 | - | - |
| MRA | 0.21 (0.04-1.16) | 0.073 | 0.15 (0.02-1.04) | 0.054 |
| MMF | 2.05 (0.47-8.92) | 0.337 | - | - |
| RASi | 0.79 (0.27-2.25) | 0.653 | - | - |
| HCQ | 0.89 (0.26-3.07) | 0.850 | - | - |
| Prednisone | 0.59 (0.17-2.06) | 0.411 | - | - |
| **Immune and inflammation (per 1 SD increase)** | | | | |
| NLR (Z-score) | 0.85 (0.49-1.47) | 0.559 | - | - |
| PLR (Z-score) | 0.97 (0.57-1.65) | 0.903 | - | - |
| SII (Z-score) | 0.75 (0.41-1.39) | 0.359 | - | - |
| SIAI (Z-score) | 0.97 (0.57-1.66) | 0.917 | - | - |
| NHR (Z-score) | 1.03 (0.60-1.77) | 0.922 | - | - |

Note: Data are presented as odds ratios (ORs) with their corresponding 95% confidence intervals (CIs). A multivariate logistic regression model was constructed to evaluate independent baseline predictors for achieving early response. Notably, variables were selected for inclusion in the final multivariate model based not only on statistical significance in the univariate analysis but also on their established clinical relevance in IgA nephropathy (e.g., age, gender, eGFR, and proteinuria). Subgroup analysis stratified by baseline proteinuria (<1.0 vs. ≥ 1.0 g/24h) showed a significantly higher ER rate in patients with higher baseline proteinuria (71.0% vs. 38.5%, Fisher's exact P = 0.018), suggesting a wider therapeutic window for patients with more pronounced baseline disease.

Abbreviations: CI, confidence interval; eGFR, estimated glomerular filtration rate; ER, early response; IgAN, IgA nephropathy; MEST-C, Oxford classification score for IgAN (M, mesangial hypercellularity; E, endocapillary hypercellularity; S, segmental glomerulosclerosis; T, tubular atrophy/interstitial fibrosis; C, cellular/fibrocellular crescents); OR, odds ratio; PRO, proteinuria.

**Supplementary Table S9. Univariate and Multivariable Predictors of Composite Clinical Remission (CR/PR) and eGFR stable (eGFR reduction < 15%).**

| Variables | Univariate Analysis HR (95% CI) | P value | Multivariable Analysis HR (95% CI) | P value |
| --- | --- | --- | --- | --- |
| Baseline characteristics | | | | |
| Age (≥ 45 vs < 45 yrs) | 0.54 (0.25–1.19) | 0.128 | 0.52 (0.23–1.18) | 0.120 |
| Gender (Male vs Female) | 1.21 (0.65–2.27) | 0.552 | 1.12 (0.58–2.15) | 0.730 |
| Baseline eGFR (< 60 vs ≥ 60 mL/min/1.73m²) | 0.80 (0.43–1.49) | 0.481 | 0.88 (0.42–1.85) | 0.735 |
| Baseline PRO (Grade) |  |  |  |  |
| Baseline PRO (＜ 0.5 g/d or g/g.Cr) | 1.00 (Reference) | — | 1.00 (Reference) | — |
| Baseline PRO (0.5 – 1.99 g/d or g/g.Cr) | 0.77 (0.31–1.89) | 0.563 | 0.82 (0.32–2.15) | 0.680 |
| Baseline PRO (2.0 - 3.5 g/d or g/g.Cr) | 0.89 (0.29–2.76) | 0.839 | 0.96 (0.31–3.05) | 0.940 |
| Baseline PRO ( > 3.5 g/d or g/g.Cr) | 1.29 (0.41–4.04) | 0.660 | 1.35 (0.38–4.80) | 0.640 |
| MEST-C score | | | | |
| M Lesion (M1 vs M0) | 1.07 (0.56–2.05) | 0.845 | 0.95 (0.43–2.05) | 0.890 |
| E Lesion (E1 vs E0) | 1.98 (1.05–3.73) | 0.035 | 2.20 (1.12–4.30) | 0.022 |
| S Lesion (S1 vs S0) | 0.79 (0.28–2.23) | 0.654 | 0.88 (0.29–2.70) | 0.820 |
| T Lesion (T1/T2 vs T0) | 1.50 (0.77–2.89) | 0.230 | 1.65 (0.82–3.40) | 0.158 |
| C Lesion (C1/C2 vs C0) | 1.50 (0.80–2.80) | 0.205 | 1.48 (0.76–2.85) | 0.250 |
| Concomitant medications | | | |  |
| SGLT2i | 1.78 (0.91–3.49) | 0.093 | 1.85 (0.90–3.80) | 0.095 |
| MRA | 0.78 (0.34–1.79) | 0.554 | — | — |
| MMF | 1.97 (0.96–4.05) | 0.065 | 1.88 (0.85–3.95) | 0.120 |
| RASi | 1.04 (0.55–1.94) | 0.912 | — | — |
| HCQ | 0.84 (0.39–1.84) | 0.669 | — | — |
| Prednisone | 0.90 (0.41–1.96) | 0.788 | — | — |
| Systemic immune-inflammation indices (per 1 SD increase) | | | |  |
| NLR | 0.80 (0.53–1.20) | 0.275 | — | — |
| PLR | 0.87 (0.64–1.18) | 0.367 | — | — |
| SII | 0.75 (0.49–1.16) | 0.195 | 0.72 (0.45–1.15) | 0.168 |
| SIAI | 0.79 (0.54–1.16) | 0.235 | — | — |
| NHR | 0.90 (0.63–1.29) | 0.573 | — | — |

**Notes:** Variables with *P* < 0.20 in the univariate analysis, alongside forced core clinical and pathological baseline covariates, were included in the multivariable model. Baseline PRO was evaluated as a categorical variable. Systemic immune-inflammation indices (NLR, PLR, SII, SIAI, NHR) were standardized via Z-score, and their ORs represent the risk per 1 Standard Deviation (SD) increase. Statistically significant values (*P* < 0.05) are highlighted in bold.

**Abbreviations:** HR, hazard ratio (derived from the proportional hazards model); CI, confidence interval; eGFR, estimated glomerular filtration rate; PRO, proteinuria; NLR, neutrophil-to-lymphocyte ratio; PLR, platelet-to-lymphocyte ratio; SII, systemic immune-inflammation index; SIAI, systemic inflammation response index; NHR, neutrophil-to-high-density lipoprotein ratio.

Supplementary Table S10. Multivariable Interaction Model for 6-Month Early Response (ER) Trajectories (**N=57**)

| Variables | β | SE | z-statistic | P-value | OR | 95% CI |
| --- | --- | --- | --- | --- | --- | --- |
| Intercept | -0.196 | 0.502 | -0.391 | 0.696 | — | — |
| Endocapillary hypercellularity (E1 vs. E0) | 0.931 | 0.713 | 1.306 | 0.192 | 2.537 | 0.627–10.264 |
| Biopsy-to-Treatment Interval, months | 0.004 | 0.005 | 0.776 | 0.438 | 1.004 | 0.994–1.014 |
| Interaction Term: E1 × Biopsy-to-treatment interval | -0.013 | 0.011 | -1.220 | 0.222 | 0.987 | 0.966–1.008 |

Note: Abbreviations: CI, confidence interval; OR, odds ratio; SE, standard error. Early Response (ER) at 6 months was defined as a ≥ 30% proportional reduction in proteinuria from baseline combined with categorical eGFR stability (decline < 15%). This multivariable logistic model evaluates the continuous cross-product interaction between the histopathological feature and temporal treatment delay across the full analytic cohort (N=57).

### Supplementary Table S11. Contingency Distribution and Early Response Rates within the Prompt-Initiation Subgroup (**≤ 3.0** Months, **n=16**)

| Variables | Non-Responders (6M ER = 0) | Responders (6M ER = 1) | Total (n) | Early Response Rate (%) |
| --- | --- | --- | --- | --- |
| **Endocapillary hypercellularity (E)** | | | | |
| E0 (Absent) | 3 | 1 | 4 | 25.0% |
| E1 (Present) | 4 | 8 | 12 | 66.7% |
| Total | 7 | 9 | 16 | 56.3% |

Note: This 2 × 2 contingency matrix isolates patients who initiated targeted-release budesonide within an immediate therapeutic window (≤ 3.0 months from tissue biopsy to drug exposure, n=16). Early response indicates clinical success achieved at the 6-month follow-up.

Supplementary Table S12. LASSO-Cox Model for 12-Month Composite Remission (N = 57).

| Variables | β | HR (95% CI) | P | Points | Clinical interpretation |
| --- | --- | --- | --- | --- | --- |
| Endocapillary hypercellularity (E) | | |  |  |  |
| E0 (Absent) | — | 1.00 (Reference) | — | 0 | Baseline risk profile. |
| E1 (Present) | 0.81 | 2.25 (1.18–4.31) | 0.014 | 100 | Acute inflammatory marker; strongest dynamic driver for accelerated mucosal remission. |
| Concomitant SGLT2i use | |  |  |  |  |
| No | — | 1.00 (Reference) | — | 0 | Standard of care regimen. |
| Yes | 0.66 | 1.93 (1.05–3.56) | 0.035 | 81 | Early hemodynamic synergistic protection; minimizes the short-term proteinuria floor effect. |
| Baseline proteinuria, g/24h | |  |  |  |  |
| ≥ 4.0 | — | 1.00 (Reference) | — | 0 | Severe initial proteinuric burden; baseline cutoff. |
| 3.0 | 0.14 | 1.15 (1.01–1.31) | 0.041 | 17 | Incremental point gain per 1 g/24h reduction. |
| 2.0 | 0.28 | 1.32 (1.02–1.62) | 0.041 | 35 | Moderate initial proteinuric burden. |
| 1.0 | 0.41 | 1.51 (1.03–1.94) | 0.041 | 52 | Mild initial proteinuric burden. |
| 0.5 | 0.48 | 1.62 (1.04–2.10) | 0.041 | 69 | Near-optimal initial proteinuric level. |

**Note:** Regression coefficients (β) and hazard ratios (HR) were derived from a LASSO-penalized multivariable Cox proportional hazards model. Nomogram points were allocated proportionally based on the regression coefficients of each predictor. **Abbreviations:** CI, confidence interval; HR, hazard ratio; LASSO, least absolute shrinkage and selection operator; SGLT2i, sodium-glucose cotransporter-2 inhibitor.

### Supplementary Table S13. Multivariable Stratified Logistic Regression Model for 3-Month Very Early Response (VER) Controlling for SGLT2i Treatment Timing.

| Variables | β | SE | z-statistic | P-value | OR | 95% CI |
| --- | --- | --- | --- | --- | --- | --- |
| Intercept | -1.386 | 1.018 | -1.361 | 0.173 | — | — |
| SGLT2i Treatment Timing | | | | | | |
| Non-user (Ref) | Ref | Ref | Ref | Ref | Ref | Ref |
| Pre-existing Stable Therapy (n=15) | 1.181 | 0.721 | 1.638 | 0.102 | 3.26 | 0.79–13.38 |
| Concurrent / New Initiation (n=19) | 1.336 | 0.699 | 1.911 | 0.056 | 3.81 | 0.97–14.98 |
| Pathology_E status (E1 vs. E0) | 0.615 | 0.599 | 1.028 | 0.304 | 1.85 | 0.57–5.98 |
| Baseline eGFR, mL/min/1.73m^2^ | 0.001 | 0.012 | 0.047 | 0.963 | 1.001 | 0.98–1.02 |
| Baseline Proteinuria, g/24h | 0.257 | 0.242 | 1.062 | 0.288 | 1.29 | 0.81–2.08 |

Abbreviations: CI, confidence interval; eGFR, estimated glomerular filtration rate; OR, odds ratio; SE, standard error; SGLT2i, sodium-glucose cotransporter-2 inhibitor. Total analytic cohort N = 57.
